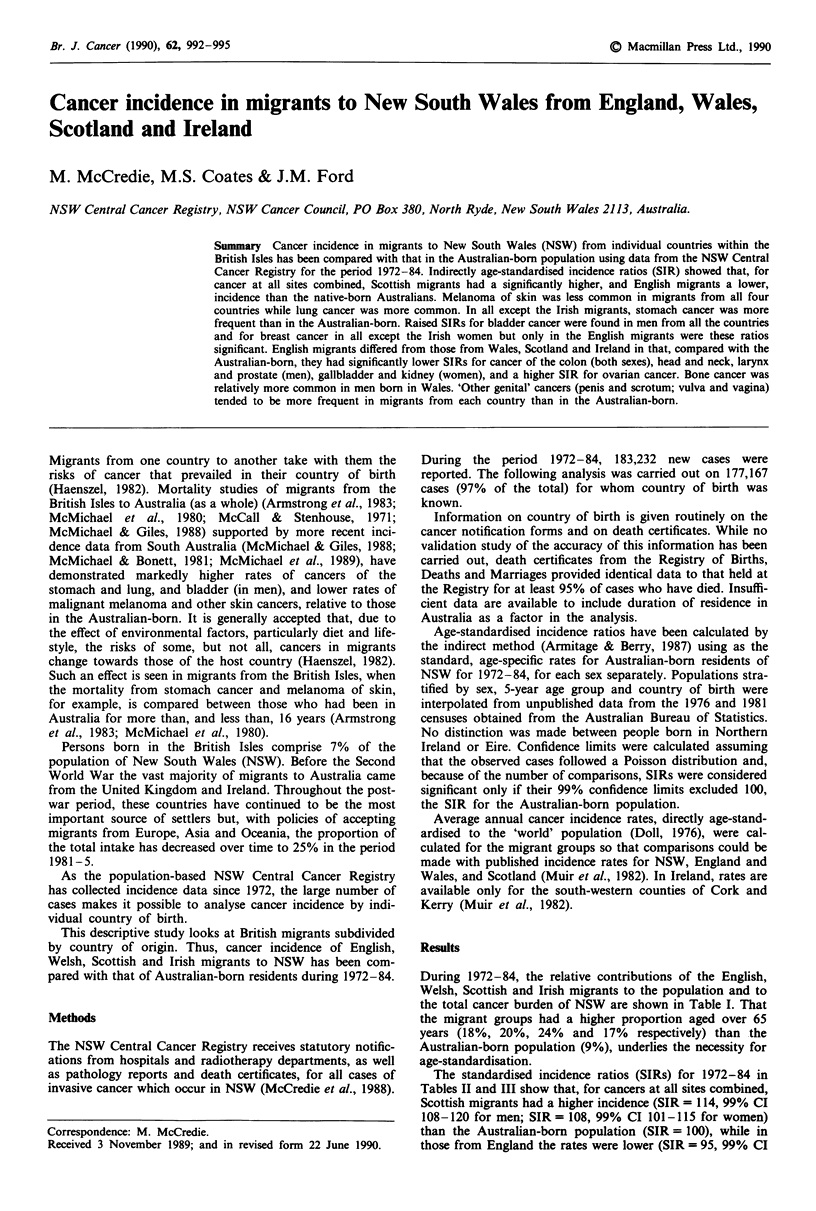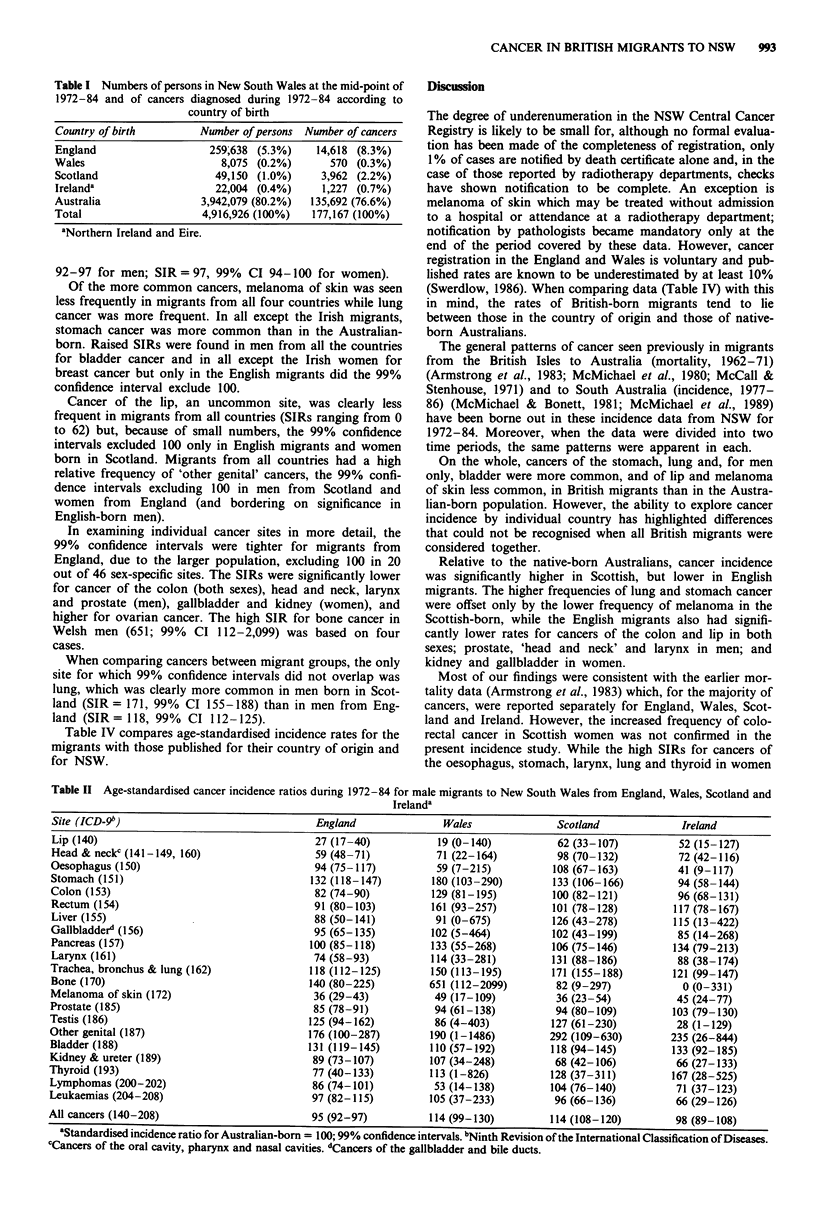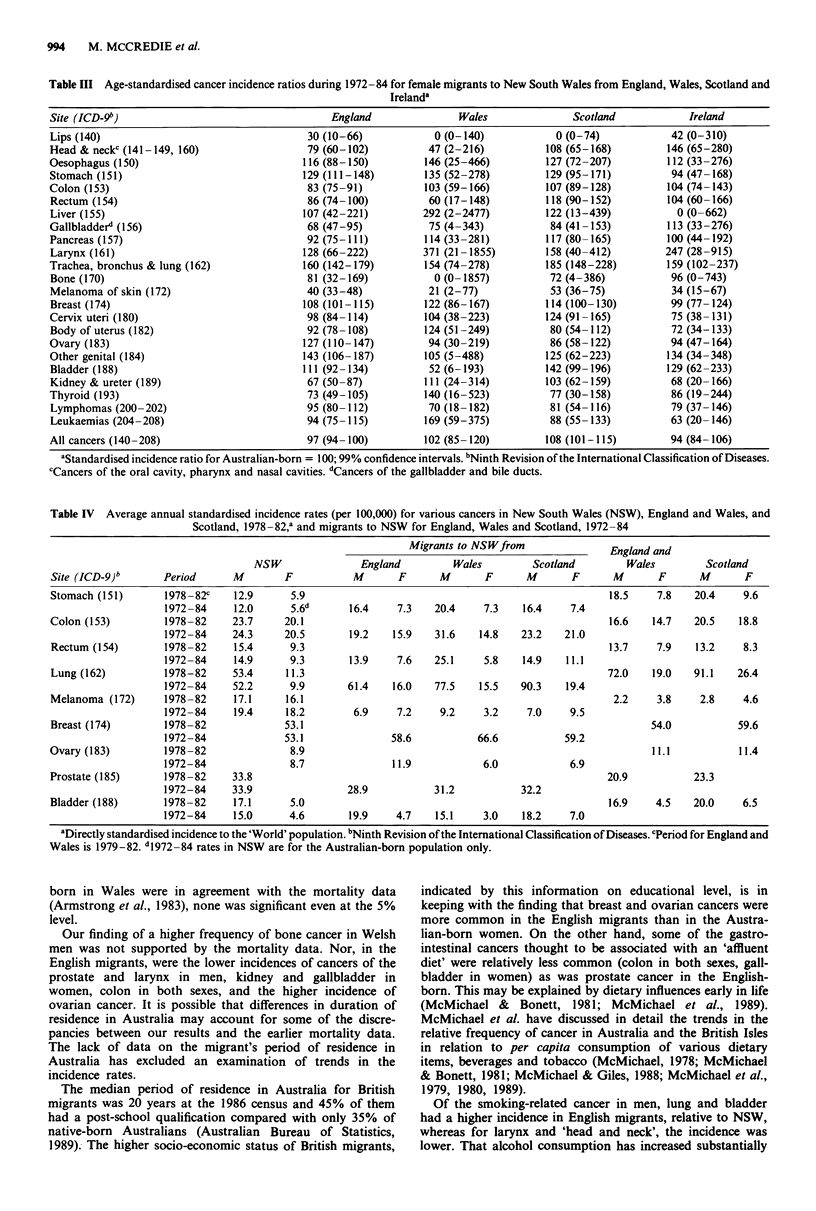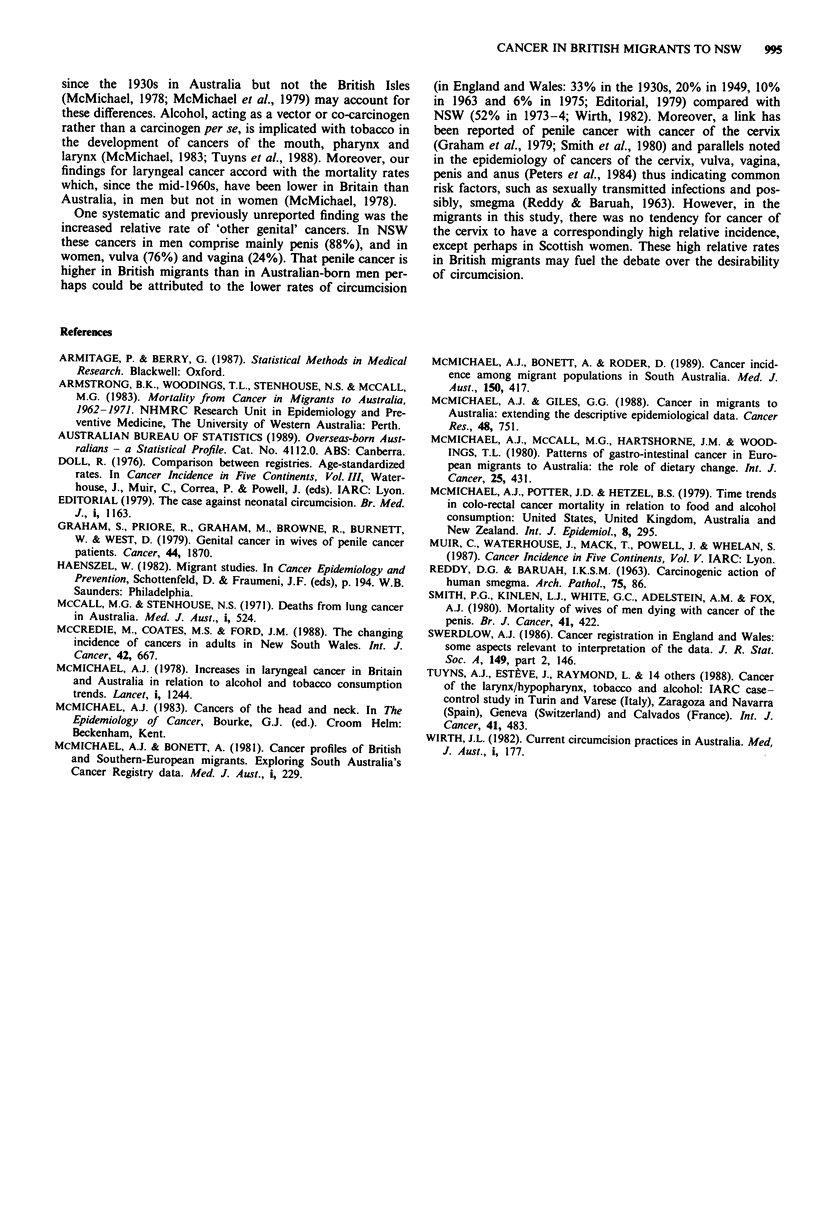# Cancer incidence in migrants to New South Wales from England, Wales, Scotland and Ireland.

**DOI:** 10.1038/bjc.1990.423

**Published:** 1990-12

**Authors:** M. McCredie, M. S. Coates, J. M. Ford

**Affiliations:** NSW Central Cancer Registry, NSW Cancer Council, Australia.

## Abstract

Cancer incidence in migrants to New South Wales (NSW) from individual countries within the British Isles has been compared with that in the Australian-born population using data from the NSW Central Cancer Registry for the period 1972-84. Indirectly age-standardised incidence ratios (SIR) showed that, for cancer at all sites combined, Scottish migrants had a significantly higher, and English migrants a lower, incidence than the native-born Australians. Melanoma of skin was less common in migrants from all four countries while lung cancer was more common. In all except the Irish migrants, stomach cancer was more frequent than in the Australian-born. Raised SIRs for bladder cancer were found in men from all the countries and for breast cancer in all except the Irish women but only in the English migrants were these ratios significant. English migrants differed from those from Wales, Scotland and Ireland in that, compared with the Australian-born, they had significantly lower SIRs for cancer of the colon (both sexes), head and neck, larynx and prostate (men), gallbladder and kidney (women), and a higher SIR for ovarian cancer. Bone cancer was relatively more common in men born in Wales. 'Other genital' cancers (penis and scrotum; vulva and vagina) tended to be more frequent in migrants from each country than in the Australian-born.


 © Macmillan Press Ltd., 1990

### Cancer incidence in migrants to New South Wales from England, Wales, Scotland and Ireland

M. McCredie, M.S. Coates & J.M. Ford

*NSW Central Cancer Registry, NSW Cancer Council, PO Box 380, North Ryde, New South Wales 2113, Australia.*

**Summary** Cancer incidence in migrants to New South Wales (NSW) from individual countries within the British Isles has been compared with that in the Australian-born population using data from the NSW Central Cancer Registry for the period 1972-84. Indirectly age-standardised incidence ratios (SIR) showed that, for cancer at all sites combined, Scottish migrants had a significantly higher, and English migrants a lower, incidence than the native-born Australians. Melanoma of skin was less common in migrants from all four countries while lung cancer was more common. In all except the Irish migrants, stomach cancer was more frequent than in the Australian-born. Raised SIRs for bladder cancer were found in men from all the countries and for breast cancer in all except the Irish women but only in the English migrants were these ratios significant. English migrants differed from those from Wales, Scotland and Ireland in that, compared with the Australian-born, they had significantly lower SIRs for cancer of the colon (both sexes), head and neck, larynx and prostate (men), gallbladder and kidney (women), and a higher SIR for ovarian cancer. Bone cancer was relatively more common in men born in Wales. 'Other genital' cancers (penis and scrotum; vulva and vagina) tended to be more frequent in migrants from each country than in the Australian-born.

Migrants from one country to another take with them the risks of cancer that prevailed in their country of birth (Haenszel, 1982). Mortality studies of migrants from the British Isles to Australia (as a whole) (Armstrong *et al.*, 1983; McMichael *et al.*, 1980; McCall & Stenhouse, 1971; McMichael & Giles, 1988) supported by more recent incidence data from South Australia (McMichael & Giles, 1988; McMichael & Bonett, 1981; McMichael *et al.*, 1989), have demonstrated markedly higher rates of cancers of the stomach and lung, and bladder (in men), and lower rates of malignant melanoma and other skin cancers, relative to those in the Australian-born. It is generally accepted that, due to the effect of environmental factors, particularly diet and life-style, the risks of some, but not all, cancers in migrants change towards those of the host country (Haenszel, 1982). Such an effect is seen in migrants from the British Isles, when the mortality from stomach cancer and melanoma of skin, for example, is compared between those who had been in Australia for more than, and less than, 16 years (Armstrong *et al.*, 1983; McMichael *et al.*, 1980).

Persons born in the British Isles comprise 7% of the population of New South Wales (NSW). Before the Second World War the vast majority of migrants to Australia came from the United Kingdom and Ireland. Throughout the post-war period, these countries have continued to be the most important source of settlers but, with policies of accepting migrants from Europe, Asia and Oceania, the proportion of the total intake has decreased over time to 25% in the period 1981-5.

As the population-based NSW Central Cancer Registry has collected incidence data since 1972, the large number of cases makes it possible to analyse cancer incidence by individual country of birth.

This descriptive study looks at British migrants subdivided by country of origin. Thus, cancer incidence of English, Welsh, Scottish and Irish migrants to NSW has been compared with that of Australian-born residents during 1972-84.

### Methods

The NSW Central Cancer Registry receives statutory notifications from hospitals and radiotherapy departments, as well as pathology reports and death certificates, for all cases of invasive cancer which occur in NSW (McCredie *et al.*, 1988).

Correspondence: M. McCredie.
Received 3 November 1989; and in revised form 22 June 1990.

During the period 1972-84, 183,232 new cases were reported. The following analysis was carried out on 177,167 cases (97% of the total) for whom country of birth was known.

Information on country of birth is given routinely on the cancer notification forms and on death certificates. While no validation study of the accuracy of this information has been carried out, death certificates from the Registry of Births, Deaths and Marriages provided identical data to that held at the Registry for at least 95% of cases who have died. Insufficient data are available to include duration of residence in Australia as a factor in the analysis.

Age-standardised incidence ratios have been calculated by the indirect method (Armitage & Berry, 1987) using as the standard, age-specific rates for Australian-born residents of NSW for 1972-84, for each sex separately. Populations stratified by sex, 5-year age group and country of birth were interpolated from unpublished data from the 1976 and 1981 censuses obtained from the Australian Bureau of Statistics. No distinction was made between people born in Northern Ireland or Eire. Confidence limits were calculated assuming that the observed cases followed a Poisson distribution and, because of the number of comparisons, SIRs were considered significant only if their 99% confidence limits excluded 100, the SIR for the Australian-born population.

Average annual cancer incidence rates, directly age-standardised to the 'world' population (Doll, 1976), were calculated for the migrant groups so that comparisons could be made with published incidence rates for NSW, England and Wales, and Scotland (Muir *et al.*, 1982). In Ireland, rates are available only for the south-western counties of Cork and Kerry (Muir *et al.*, 1982).

### Results

During 1972-84, the relative contributions of the English, Welsh, Scottish and Irish migrants to the population and to the total cancer burden of NSW are shown in Table I. That the migrant groups had a higher proportion aged over 65 years (18%, 20%, 24% and 17% respectively) than the Australian-born population (9%), underlies the necessity for age-standardisation.

The standardised incidence ratios (SIRs) for 1972-84 in Tables II and III show that, for cancers at all sites combined, Scottish migrants had a higher incidence (SIR = 114, 99% CI 108-120 for men; SIR = 108, 99% CI 101-115 for women) than the Australian-born population (SIR = 100), while in those from England the rates were lower (SIR = 95, 99% CI

**Table I** Numbers of persons in New South Wales at the mid-point of 1972–84 and of cancers diagnosed during 1972–84 according to country of birth

| *Country of birth* | *Number of persons* | *Number of cancers* |
|---|---|---|
| England | 259,638 (5.3%) | 14,618 (8.3%) |
| Wales | 8,075 (0.2%) | 570 (0.3%) |
| Scotland | 49,150 (1.0%) | 3,962 (2.2%) |
| Ireland[a] | 22,004 (0.4%) | 1,227 (0.7%) |
| Australia | 3,942,079 (80.2%) | 135,692 (76.6%) |
| Total | 4,916,926 (100%) | 177,167 (100%) |

[a]Northern Ireland and Eire.

92–97 for men; SIR = 97, 99% CI 94–100 for women).

Of the more common cancers, melanoma of skin was seen less frequently in migrants from all four countries while lung cancer was more frequent. In all except the Irish migrants, stomach cancer was more common than in the Australian-born. Raised SIRs were found in men from all the countries for bladder cancer and in all except the Irish women for breast cancer but only in the English migrants did the 99% confidence interval exclude 100.

Cancer of the lip, an uncommon site, was clearly less frequent in migrants from all countries (SIRs ranging from 0 to 62) but, because of small numbers, the 99% confidence intervals excluded 100 only in English migrants and women born in Scotland. Migrants from all countries had a high relative frequency of 'other genital' cancers, the 99% confidence intervals excluding 100 in men from Scotland and women from England (and bordering on significance in English-born men).

In examining individual cancer sites in more detail, the 99% confidence intervals were tighter for migrants from England, due to the larger population, excluding 100 in 20 out of 46 sex-specific sites. The SIRs were significantly lower for cancer of the colon (both sexes), head and neck, larynx and prostate (men), gallbladder and kidney (women), and higher for ovarian cancer. The high SIR for bone cancer in Welsh men (651; 99% CI 112–2,099) was based on four cases.

When comparing cancers between migrant groups, the only site for which 99% confidence intervals did not overlap was lung, which was clearly more common in men born in Scotland (SIR = 171, 99% CI 155–188) than in men from England (SIR = 118, 99% CI 112–125).

Table IV compares age-standardised incidence rates for the migrants with those published for their country of origin and for NSW.

### Discussion

The degree of underenumeration in the NSW Central Cancer Registry is likely to be small for, although no formal evaluation has been made of the completeness of registration, only 1% of cases are notified by death certificate alone and, in the case of those reported by radiotherapy departments, checks have shown notification to be complete. An exception is melanoma of skin which may be treated without admission to a hospital or attendance at a radiotherapy department; notification by pathologists became mandatory only at the end of the period covered by these data. However, cancer registration in the England and Wales is voluntary and published rates are known to be underestimated by at least 10% (Swerdlow, 1986). When comparing data (Table IV) with this in mind, the rates of British-born migrants tend to lie between those in the country of origin and those of native-born Australians.

The general patterns of cancer seen previously in migrants from the British Isles to Australia (mortality, 1962–71) (Armstrong *et al.*, 1983; McMichael *et al.*, 1980; McCall & Stenhouse, 1971) and to South Australia (incidence, 1977–86) (McMichael & Bonett, 1981; McMichael *et al.*, 1989) have been borne out in these incidence data from NSW for 1972–84. Moreover, when the data were divided into two time periods, the same patterns were apparent in each.

On the whole, cancers of the stomach, lung and, for men only, bladder were more common, and of lip and melanoma of skin less common, in British migrants than in the Australian-born population. However, the ability to explore cancer incidence by individual country has highlighted differences that could not be recognised when all British migrants were considered together.

Relative to the native-born Australians, cancer incidence was significantly higher in Scottish, but lower in English migrants. The higher frequencies of lung and stomach cancer were offset only by the lower frequency of melanoma in the Scottish-born, while the English migrants also had significantly lower rates for cancers of the colon and lip in both sexes; prostate, 'head and neck' and larynx in men; and kidney and gallbladder in women.

Most of our findings were consistent with the earlier mortality data (Armstrong *et al.*, 1983) which, for the majority of cancers, were reported separately for England, Wales, Scotland and Ireland. However, the increased frequency of colorectal cancer in Scottish women was not confirmed in the present incidence study. While the high SIRs for cancers of the oesophagus, stomach, larynx, lung and thyroid in women

**Table II** Age-standardised cancer incidence ratios during 1972–84 for male migrants to New South Wales from England, Wales, Scotland and Ireland[a]

| *Site (ICD-9[b])* | *England* | *Wales* | *Scotland* | *Ireland* |
|---|---|---|---|---|
| Lip (140) | 27 (17–40) | 19 (0–140) | 62 (33–107) | 52 (15–127) |
| Head & neck[c] (141–149, 160) | 59 (48–71) | 71 (22–164) | 98 (70–132) | 72 (42–116) |
| Oesophagus (150) | 94 (75–117) | 59 (7–215) | 108 (67–163) | 41 (9–117) |
| Stomach (151) | 132 (118–147) | 180 (103–290) | 133 (106–166) | 94 (58–144) |
| Colon (153) | 82 (74–90) | 129 (81–195) | 100 (82–121) | 96 (68–131) |
| Rectum (154) | 91 (80–103) | 161 (93–257) | 101 (78–128) | 117 (78–167) |
| Liver (155) | 88 (50–141) | 91 (0–675) | 126 (43–278) | 115 (13–422) |
| Gallbladder[d] (156) | 95 (65–135) | 102 (5–464) | 102 (43–199) | 85 (14–268) |
| Pancreas (157) | 100 (85–118) | 133 (55–268) | 106 (75–146) | 134 (79–213) |
| Larynx (161) | 74 (58–93) | 114 (33–281) | 131 (88–186) | 88 (38–174) |
| Trachea, bronchus & lung (162) | 118 (112–125) | 150 (113–195) | 171 (155–188) | 121 (99–147) |
| Bone (170) | 140 (80–225) | 651 (112–2099) | 82 (9–297) | 0 (0–331) |
| Melanoma of skin (172) | 36 (29–43) | 49 (17–109) | 36 (23–54) | 45 (24–77) |
| Prostate (185) | 85 (78–91) | 94 (61–138) | 94 (80–109) | 103 (79–130) |
| Testis (186) | 125 (94–162) | 86 (4–403) | 127 (61–230) | 28 (1–129) |
| Other genital (187) | 176 (100–287) | 190 (1–1486) | 292 (109–630) | 235 (26–844) |
| Bladder (188) | 131 (119–145) | 110 (57–192) | 118 (94–145) | 133 (92–185) |
| Kidney & ureter (189) | 89 (73–107) | 107 (34–248) | 68 (42–106) | 66 (27–133) |
| Thyroid (193) | 77 (40–133) | 113 (1–826) | 128 (37–311) | 167 (28–525) |
| Lymphomas (200–202) | 86 (74–101) | 53 (14–138) | 104 (76–140) | 71 (37–123) |
| Leukaemias (204–208) | 97 (82–115) | 105 (37–233) | 96 (66–136) | 66 (29–126) |
| All cancers (140–208) | 95 (92–97) | 114 (99–130) | 114 (108–120) | 98 (89–108) |

[a]Standardised incidence ratio for Australian-born = 100; 99% confidence intervals. [b]Ninth Revision of the International Classification of Diseases. [c]Cancers of the oral cavity, pharynx and nasal cavities. [d]Cancers of the gallbladder and bile ducts.

**Table III** Age-standardised cancer incidence ratios during 1972–84 for female migrants to New South Wales from England, Wales, Scotland and Ireland[a]

| *Site (ICD-9[b])* | *England* | *Wales* | *Scotland* | *Ireland* |
|---|---|---|---|---|
| Lips (140) | 30 (10–66) | 0 (0–140) | 0 (0–74) | 42 (0–310) |
| Head & neck[c] (141–149, 160) | 79 (60–102) | 47 (2–216) | 108 (65–168) | 146 (65–280) |
| Oesophagus (150) | 116 (88–150) | 146 (25–466) | 127 (72–207) | 112 (33–276) |
| Stomach (151) | 129 (111–148) | 135 (52–278) | 129 (95–171) | 94 (47–168) |
| Colon (153) | 83 (75–91) | 103 (59–166) | 107 (89–128) | 104 (74–143) |
| Rectum (154) | 86 (74–100) | 60 (17–148) | 118 (90–152) | 104 (60–166) |
| Liver (155) | 107 (42–221) | 292 (2–2477) | 122 (13–439) | 0 (0–662) |
| Gallbladder[d] (156) | 68 (47–95) | 75 (4–343) | 84 (41–153) | 113 (33–276) |
| Pancreas (157) | 92 (75–111) | 114 (33–281) | 117 (80–165) | 100 (44–192) |
| Larynx (161) | 128 (66–222) | 371 (21–1855) | 158 (40–412) | 247 (28–915) |
| Trachea, bronchus & lung (162) | 160 (142–179) | 154 (74–278) | 185 (148–228) | 159 (102–237) |
| Bone (170) | 81 (32–169) | 0 (0–1857) | 72 (4–386) | 96 (0–743) |
| Melanoma of skin (172) | 40 (33–48) | 21 (2–77) | 53 (36–75) | 34 (15–67) |
| Breast (174) | 108 (101–115) | 122 (86–167) | 114 (100–130) | 99 (77–124) |
| Cervix uteri (180) | 98 (84–114) | 104 (38–223) | 124 (91–165) | 75 (38–131) |
| Body of uterus (182) | 92 (78–108) | 124 (51–249) | 80 (54–112) | 72 (34–133) |
| Ovary (183) | 127 (110–147) | 94 (30–219) | 86 (58–122) | 94 (47–164) |
| Other genital (184) | 143 (106–187) | 105 (5–488) | 125 (62–223) | 134 (34–348) |
| Bladder (188) | 111 (92–134) | 52 (6–193) | 142 (99–196) | 129 (62–233) |
| Kidney & ureter (189) | 67 (50–87) | 111 (24–314) | 103 (62–159) | 68 (20–166) |
| Thyroid (193) | 73 (49–105) | 140 (16–523) | 77 (30–158) | 86 (19–244) |
| Lymphomas (200–202) | 95 (80–112) | 70 (18–182) | 81 (54–116) | 79 (37–146) |
| Leukaemias (204–208) | 94 (75–115) | 169 (59–375) | 88 (55–133) | 63 (20–146) |
| All cancers (140–208) | 97 (94–100) | 102 (85–120) | 108 (101–115) | 94 (84–106) |

[a]Standardised incidence ratio for Australian-born = 100; 99% confidence intervals. [b]Ninth Revision of the International Classification of Diseases. [c]Cancers of the oral cavity, pharynx and nasal cavities. [d]Cancers of the gallbladder and bile ducts.

**Table IV** Average annual standardised incidence rates (per 100,000) for various cancers in New South Wales (NSW), England and Wales, and Scotland, 1978–82,[a] and migrants to NSW for England, Wales and Scotland, 1972–84

| | | | | *Migrants to NSW from* | | | | | | *England and Wales* | | *Scotland* | |
|---|---|---|---|---|---|---|---|---|---|---|---|---|---|
| | | *NSW* | | *England* | | *Wales* | | *Scotland* | | | | | |
| *Site (ICD-9)[b]* | *Period* | *M* | *F* | *M* | *F* | *M* | *F* | *M* | *F* | *M* | *F* | *M* | *F* |
| Stomach (151) | 1978–82[c] | 12.9 | 5.9 | | | | | | | 18.5 | 7.8 | 20.4 | 9.6 |
| | 1972–84 | 12.0 | 5.6[d] | 16.4 | 7.3 | 20.4 | 7.3 | 16.4 | 7.4 | | | | |
| Colon (153) | 1978–82 | 23.7 | 20.1 | | | | | | | 16.6 | 14.7 | 20.5 | 18.8 |
| | 1972–84 | 24.3 | 20.5 | 19.2 | 15.9 | 31.6 | 14.8 | 23.2 | 21.0 | | | | |
| Rectum (154) | 1978–82 | 15.4 | 9.3 | | | | | | | 13.7 | 7.9 | 13.2 | 8.3 |
| | 1972–84 | 14.9 | 9.3 | 13.9 | 7.6 | 25.1 | 5.8 | 14.9 | 11.1 | | | | |
| Lung (162) | 1978–82 | 53.4 | 11.3 | | | | | | | 72.0 | 19.0 | 91.1 | 26.4 |
| | 1972–84 | 52.2 | 9.9 | 61.4 | 16.0 | 77.5 | 15.5 | 90.3 | 19.4 | | | | |
| Melanoma (172) | 1978–82 | 17.1 | 16.1 | | | | | | | 2.2 | 3.8 | 2.8 | 4.6 |
| | 1972–84 | 19.4 | 18.2 | 6.9 | 7.2 | 9.2 | 3.2 | 7.0 | 9.5 | | | | |
| Breast (174) | 1978–82 | | 53.1 | | | | | | | | 54.0 | | 59.6 |
| | 1972–84 | | 53.1 | | 58.6 | | 66.6 | | 59.2 | | | | |
| Ovary (183) | 1978–82 | | 8.9 | | | | | | | | 11.1 | | 11.4 |
| | 1972–84 | | 8.7 | | 11.9 | | 6.0 | | 6.9 | | | | |
| Prostate (185) | 1978–82 | 33.8 | | | | | | | | 20.9 | | 23.3 | |
| | 1972–84 | 33.9 | | 28.9 | | 31.2 | | 32.2 | | | | | |
| Bladder (188) | 1978–82 | 17.1 | 5.0 | | | | | | | 16.9 | 4.5 | 20.0 | 6.5 |
| | 1972–84 | 15.0 | 4.6 | 19.9 | 4.7 | 15.1 | 3.0 | 18.2 | 7.0 | | | | |

[a]Directly standardised incidence to the 'World' population. [b]Ninth Revision of the International Classification of Diseases. [c]Period for England and Wales is 1979–82. [d]1972–84 rates in NSW are for the Australian-born population only.

born in Wales were in agreement with the mortality data (Armstrong *et al.*, 1983), none was significant even at the 5% level.

Our finding of a higher frequency of bone cancer in Welsh men was not supported by the mortality data. Nor, in the English migrants, were the lower incidences of cancers of the prostate and larynx in men, kidney and gallbladder in women, colon in both sexes, and the higher incidence of ovarian cancer. It is possible that differences in duration of residence in Australia may account for some of the discrepancies between our results and the earlier mortality data. The lack of data on the migrant's period of residence in Australia has excluded an examination of trends in the incidence rates.

The median period of residence in Australia for British migrants was 20 years at the 1986 census and 45% of them had a post-school qualification compared with only 35% of native-born Australians (Australian Bureau of Statistics, 1989). The higher socio-economic status of British migrants, indicated by this information on educational level, is in keeping with the finding that breast and ovarian cancers were more common in the English migrants than in the Australian-born women. On the other hand, some of the gastrointestinal cancers thought to be associated with an 'affluent diet' were relatively less common (colon in both sexes, gallbladder in women) as was prostate cancer in the English-born. This may be explained by dietary influences early in life (McMichael & Bonett, 1981; McMichael *et al.*, 1989). McMichael *et al.* have discussed in detail the trends in the relative frequency of cancer in Australia and the British Isles in relation to *per capita* consumption of various dietary items, beverages and tobacco (McMichael, 1978; McMichael & Bonett, 1981; McMichael & Giles, 1988; McMichael *et al.*, 1979, 1980, 1989).

Of the smoking-related cancer in men, lung and bladder had a higher incidence in English migrants, relative to NSW, whereas for larynx and 'head and neck', the incidence was lower. That alcohol consumption has increased substantially since the 1930s in Australia but not the British Isles (McMichael, 1978; McMichael *et al.*, 1979) may account for these differences. Alcohol, acting as a vector or co-carcinogen rather than a carcinogen *per se*, is implicated with tobacco in the development of cancers of the mouth, pharynx and larynx (McMichael, 1983; Tuyns *et al.*, 1988). Moreover, our findings for laryngeal cancer accord with the mortality rates which, since the mid-1960s, have been lower in Britain than Australia, in men but not in women (McMichael, 1978).

One systematic and previously unreported finding was the increased relative rate of 'other genital' cancers. In NSW these cancers in men comprise mainly penis (88%), and in women, vulva (76%) and vagina (24%). That penile cancer is higher in British migrants than in Australian-born men perhaps could be attributed to the lower rates of circumcision (in England and Wales: 33% in the 1930s, 20% in 1949, 10% in 1963 and 6% in 1975; Editorial, 1979) compared with NSW (52% in 1973–4; Wirth, 1982). Moreover, a link has been reported of penile cancer with cancer of the cervix (Graham *et al.*, 1979; Smith *et al.*, 1980) and parallels noted in the epidemiology of cancers of the cervix, vulva, vagina, penis and anus (Peters *et al.*, 1984) thus indicating common risk factors, such as sexually transmitted infections and possibly, smegma (Reddy & Baruah, 1963). However, in the migrants in this study, there was no tendency for cancer of the cervix to have a correspondingly high relative incidence, except perhaps in Scottish women. These high relative rates in British migrants may fuel the debate over the desirability of circumcision.

### References

ARMITAGE, P. & BERRY, G. (1987). *Statistical Methods in Medical Research*. Blackwell: Oxford.

ARMSTRONG, B.K., WOODINGS, T.L., STENHOUSE, N.S. & MCCALL, M.G. (1983). *Mortality from Cancer in Migrants to Australia, 1962–1971*. NHMRC Research Unit in Epidemiology and Preventive Medicine, The University of Western Australia: Perth.

AUSTRALIAN BUREAU OF STATISTICS (1989). *Overseas-born Australians – a Statistical Profile*. Cat. No. 4112.0. ABS: Canberra.

DOLL, R. (1976). Comparison between registries. Age-standardized rates. In *Cancer Incidence in Five Continents, Vol. III*, Waterhouse, J., Muir, C., Correa, P. & Powell, J. (eds). IARC: Lyon.

EDITORIAL (1979). The case against neonatal circumcision. *Br. Med. J.*, **i**, 1163.

GRAHAM, S., PRIORE, R., GRAHAM, M., BROWNE, R., BURNETT, W. & WEST, D. (1979). Genital cancer in wives of penile cancer patients. *Cancer*, **44**, 1870.

HAENSZEL, W. (1982). Migrant studies. In *Cancer Epidemiology and Prevention*, Schottenfeld, D. & Fraumeni, J.F. (eds), p. 194. W.B. Saunders: Philadelphia.

MCCALL, M.G. & STENHOUSE, N.S. (1971). Deaths from lung cancer in Australia. *Med. J. Aust.*, **i**, 524.

MCCREDIE, M., COATES, M.S. & FORD, J.M. (1988). The changing incidence of cancers in adults in New South Wales. *Int. J. Cancer*, **42**, 667.

MCMICHAEL, A.J. (1978). Increases in laryngeal cancer in Britain and Australia in relation to alcohol and tobacco consumption trends. *Lancet*, **i**, 1244.

MCMICHAEL, A.J. (1983). Cancers of the head and neck. In *The Epidemiology of Cancer*, Bourke, G.J. (ed.). Croom Helm: Beckenham, Kent.

MCMICHAEL, A.J. & BONETT, A. (1981). Cancer profiles of British and Southern-European migrants. Exploring South Australia's Cancer Registry data. *Med. J. Aust.*, **i**, 229.

MCMICHAEL, A.J., BONETT, A. & RODER, D. (1989). Cancer incidence among migrant populations in South Australia. *Med. J. Aust.*, **150**, 417.

MCMICHAEL, A.J. & GILES, G.G. (1988). Cancer in migrants to Australia: extending the descriptive epidemiological data. *Cancer Res.*, **48**, 751.

MCMICHAEL, A.J., MCCALL, M.G., HARTSHORNE, J.M. & WOODINGS, T.L. (1980). Patterns of gastro-intestinal cancer in European migrants to Australia: the role of dietary change. *Int. J. Cancer*, **25**, 431.

MCMICHAEL, A.J., POTTER, J.D. & HETZEL, B.S. (1979). Time trends in colo-rectal cancer mortality in relation to food and alcohol consumption: United States, United Kingdom, Australia and New Zealand. *Int. J. Epidemiol.*, **8**, 295.

MUIR, C., WATERHOUSE, J., MACK, T., POWELL, J. & WHELAN, S. (1987). *Cancer Incidence in Five Continents, Vol. V*. IARC: Lyon.

REDDY, D.G. & BARUAH, I.K.S.M. (1963). Carcinogenic action of human smegma. *Arch. Pathol.*, **75**, 86.

SMITH, P.G., KINLEN, L.J., WHITE, G.C., ADELSTEIN, A.M. & FOX, A.J. (1980). Mortality of wives of men dying with cancer of the penis. *Br. J. Cancer*, **41**, 422.

SWERDLOW, A.J. (1986). Cancer registration in England and Wales: some aspects relevant to interpretation of the data. *J. R. Stat. Soc. A*, **149**, part 2, 146.

TUYNS, A.J., ESTÈVE, J., RAYMOND, L. & 14 others (1988). Cancer of the larynx/hypopharynx, tobacco and alcohol: IARC case–control study in Turin and Varese (Italy), Zaragoza and Navarra (Spain), Geneva (Switzerland) and Calvados (France). *Int. J. Cancer*, **41**, 483.

WIRTH, J.L. (1982). Current circumcision practices in Australia. *Med, J. Aust.*, **i**, 177.